# Johanson-Blizzard syndrome

**DOI:** 10.1590/S1808-86942010000600020

**Published:** 2015-10-19

**Authors:** Sérgio Ramos, Henrique F. Ramos, Rosangela F. Ramos, Carlos A.M. Peixoto, Bernardo F. Ramos

**Affiliations:** 1 Full Professor of Otorhinolaryngology - Health Sciences Center - Federal University of Espirito Santo (UFES). Head of the Specialized Medicine - Health Sciences Center - Federal University of Espirito Santo (UFES) and Head of the ENT Department - Cassiano Antônio de Moraes University Hospital (HUCAM); 2 MD. ENT - ABORL-CCF. Preceptor at the ENT Clinic - University of São Paulo Medical School (FMUSP); 3 MSc in ENT and PhD in Medicine - Federal University of São Paulo Medical School (EPM-UNIFESP). MD; ENT in Vitória, ES; 4 MD, Neuropediatrician - Cassiano Antônio de Moraes University Hospital (HUCAM); 5 ENT Resident - University of São Paulo Medical School (FMUSP). Serviço de Otorrinolaringologia do Hospital Universitário “Cassiano Antonio de Moraes” - Centro de Ciências da Saúde da Universidade Federal do Espírito Santo

**Keywords:** congenital abnormalities, craniofacial abnormalities, otitis media with effusion, hearing loss

## INTRODUCTION AND LITERATURE REVIEW

The Johanson-Blizzard Syndrome (JBS) is hereditary autosomal recessive. It bears ectodermal dysplasia, endocrine and exocrine failure, and there can be mental and development failure. It is marked by nasal wing hypoplasia or aplasia and dental abnormalities^1^. There may be prenatal growth failure; sensorineural hearing loss; hypotonia; microcephaly; skull mid-line defects; less hair or frontal toupet; nasolacrimal fistula; calicectasia or hydronephrosis; anal imperforation; septal vagina; cryptorchidism; micropenis; primary hypothyroidism; pancreatic failure with malabsorption. First described in 1971 by Johanson & Blizzard^2^. There were 51 cases reported in the literature by 2004.

## CASE DESCRIPTION

**Figure 1:**
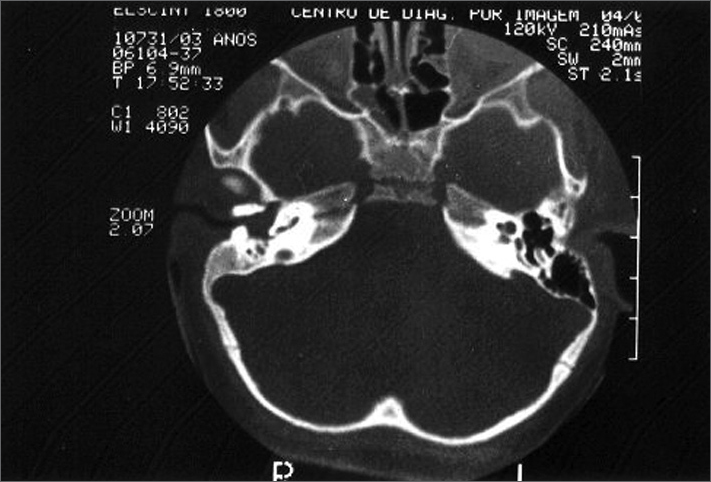
Axial view of a temporal bone CT scan: tapering external ear canal atresia, not reaching what would be the tympanic membranes. No mastoid antrum cell aeration and tympanic cavity filled by soft tissue in the right ear.

A three-year old Caucasian female, with development retardation. Born at the 8^th^ month of gestation, out of a C-section, she was discharged from the hospital after three days. At four months of age she could not support her own head, when she was submitted to surgery to correct craniostenosis. She managed to seat with support at 12 months of age and without support at eighteen. She could not pronounce words and it seemed she was also unable to hear. She could stay standing with support but could not walk. She had undergone an uneventful gestation. PHYSICAL EXAM: hypertelorism; thin nose; micrognathia; global hypotonia and ligament laxity; level V muscle strength in the four limbs; myotactic hyporeflexia; normal motor coordination in the upper and lower limbs; plantar skin reflex in bilateral flexion. Large palatine tonsils, ear pinna with altered helixes; external ear canal (EEC) atresia and nasal wing hypoplasia. COMPLEMENTARY TESTS: Normal thyroid hormones. BERA: Right Ear - no auditory evoked potentials with 130 dBpeSpl; Left Ear - threshold at 100 dBpeSpl. Wave morphology analysis and latency differentials I-III, III-V and I-V (11PPS): Left ear - 130 dBpeSpl, waves I, II and III of normal morphology; wave V with lower amplitude in twice that of wave I; normal latency interval. TEMPORAL BONE CT SCAN: EEC atresia all the way to the middle ears and presence of material resembling soft tissue; normal middle ears, inner ears and inner acoustic meatus; except for aeration loss of the mastoid antrum and cells and right ear tympanic cavity filled with soft tissue material (Fig. 1).

## DISCUSSION

The cases considered JBS, have a variety of clinical and anatomical findings differing a little from the original description. In this patient there is no hypothyroidism - present in 1/3 of the cases^1^, ^3^ and pancreatic failure was not investigated because she did not have symptoms which would justify it. There are many cases in which pancreatic failure was not mentioned because the diagnosis was intrauterine, or because the focus was the radiological study of the temporal bone^4^. There are cases of siblings with JBS^5^ and consanguinity is considered a risk factor, and there are doubts concerning the recessive autosomal inheritance. In most of the cases there was no genetic investigation and there are well defined cases with normal cariotype^6^.

EEC malformation in this syndrome had not been described. It was only in one case that there was a CT scan^4^ in which there was bilateral cystic dilatation of the cochlea and vestibule. There are no reports of secretory otitis media, which was possible upon CT scan. The malformation did not allow for otoscopy and tympanometry.

## FINAL REMARKS

Early diagnosis is important because of pancreatic failure and hypothyroidism, which can be the main risk factors for mental retardation^3^. In the prenatal ultrasound scan, the sigmoid was dilated and there was nasal wing aplasia (beak-shaped nose), which may represent the earlier diagnosis in JBS, especially in gestations of consanguineous couples^7^. In relation to sensorineural hearing loss, one must consider rehabilitation with a personal hearing amplification device and cochlear implant. As to conduction hearing loss, clinical treatment must be considered.
